# Psychometric Properties and Validation of the General Anxiety Disorder 7-Item Scale Among Adolescents With Persistent Post-Concussive Symptoms

**DOI:** 10.1089/neur.2022.0075

**Published:** 2023-04-27

**Authors:** Lyscha A. Marcynyszyn, Carolyn A. McCarty, Sara P.D. Chrisman, Douglas F. Zatzick, Ashleigh M. Johnson, Jin Wang, Robert J. Hilt, Frederick P. Rivara

**Affiliations:** ^1^ ICF International, Reston, Virginia, USA.; ^2^ Center for Child Health, Behavior, and Development, Seattle Children's Research Institute, Seattle, Washington, USA.; ^3^ Harborview Medical Center, University of Washington School of Medicine, Department of Epidemiology, School of Public Health, University of Washington, Seattle, Washington, USA.; ^4^ College of Health and Human Services, School of Exercise and Nutritional Sciences, San Diego State University, San Diego, California, USA.; ^5^ Harborview Injury Prevention and Research Center, Department of Pediatrics, Department of Epidemiology, School of Public Health, University of Washington, Seattle, Washington, USA.; ^6^ Psychiatry and Behavioral Sciences, University of Washington, Seattle Children's Hospital, Department of Epidemiology, School of Public Health, University of Washington, Seattle, Washington, USA.; ^7^ Department of Pediatrics, Department of Epidemiology, School of Public Health, University of Washington, Seattle, Washington, USA.

**Keywords:** adolescents, anxiety, concussion, psychometrics, traumatic brain injury

## Abstract

The General Anxiety Disorder 7-Item (GAD-7) scale is commonly used in primary care as a self-report measure of general anxiety symptoms with adult populations. There is little psychometric research on this measure with adolescent populations, particularly those with persistent post-concussive symptoms (PPCS). This study examined the psychometrics properties of the GAD-7 among youth with PPCS. We used baseline data from a randomized controlled trial of collaborative care for treatment of PPCS among 200 sports-injured adolescents 11–18 years of age (M_age_ = 14.7 years, standard deviation = 1.7). Eligible adolescents had three or more PPCS that lasted for ≥1 month and spoke English. Adolescents reported on their anxious (GAD-7 and Revised Child Anxiety and Depression Scale–Short Version [anxiety subscale]; RCADS) and depressive (Patient Health Questionnaire-9; PHQ-9) symptoms. Parents used the RCADS to report on their adolescents' anxious symptoms. The GAD-7 had good internal validity (Cronbach's alpha = 0.87), and significant (*p* < 0.001) correlations were detected between the GAD-7 and youth and parent report of anxiety on RCADS (*r* = 0.73 and *r* = 0.29) and PHQ-9 (*r* = 0.77) scores. Confirmatory factor analysis suggested a one-factor solution. These results suggest that the GAD-7 is a valid measure of anxiety with good psychometric properties for youth experiencing PPCS. ClinicalTrials.gov identifier: NCT03034720

## Introduction

Concussion is increasingly common among U.S. children and adolescents, affecting up to 1.9 million youth annually.^1^ Though concussion symptoms such as headache, fatigue, difficulty concentrating, and sleep disruption typically resolve within days to weeks post-injury, an estimated 20–30% of youth experience persistent post-concussive symptoms (PPCS) defined as impairment lasting longer than 1 month.^2–4^ For these youth, the period subsequent to a concussion is associated with particularly high risk for the development of anxious and depressive symptoms,^5–7^ which can lead to disengagement within school and community settings^5,8,9^ and the disruption of school performance and peer relationships.^10^ The high incidence of anxious and depressive symptoms is also associated with greater risk for suicidality.^11–13^ In addition to the linkages between PPCS and anxious and depressive outcomes, these internalizing symptoms are also risk factors for the occurrence of PPCS.^7,14–17^

As described by Mossman and colleagues,^18^ few self-report measures of adolescent anxiety exist with demonstrated validity and reliability. Further, we are unaware of any publications that investigate the psychometric properties of this standardized measure of anxiety in a sample of youth with PPCS. Recent studies^18,19^ support the use of the 7-item Generalized Anxiety Disorder Scale (GAD-7) as a valid measure of adolescent anxious symptomatology in non-clinical and clinical (adolescents with generalized anxiety disorder [GAD]) samples, respectively. The GAD-7 was developed by Spitzer and colleagues^20^ as a self-report screening measure for GAD to increase detection of this disorder in adults in primary care settings. Overall, earlier studies with non-psychiatric samples that assessed the structural validity of the GAD-7 revealed a unidimensional factor structure.^19,21,22^ However, the psychometric properties of the scale have yet to be evaluated in samples of youth with PPCS.

This study had three overarching aims within this sample of youth with PPCS. First, we assessed the reliability, factorial validity, and construct validity of the GAD-7. Second, we examined the relationship between adolescents' report on their own anxiety, using the GAD-7 and the Revised Child Anxiety and Depression Scale–Short Version (RCADS), and parents' report on their children's anxiety using the RCADS as an indicator of convergent validity. Third, given the heterogeneity of the PPCS patient population, we will also examine severity and potential differential validity of the GAD-7 by demographic factors (age and gender) and history of past trauma or mental health issues. Specifically, we sought to determine whether the mean scores and Cronbach's alphas for the GAD-7 differed on the basis of gender (female vs. male), age (11–13 vs. 14–15 vs. 16–18), past anxiety, previous mental health problems (defined as depression, attention deficit hyperactivity disorder or attention deficit disorder diagnosis, or other mental health problems), trauma history, and current depressive symptoms.

## Methods

### Participants

This study used baseline data from a randomized comparative effectiveness trial of a collaborative care model for treatment of PPCS among adolescents 11–18 years of age with sports-related concussions. Adolescents and their parents were recruited from specialty pediatric clinics throughout western Washington.^23,24^ To be eligible, adolescents were required to have three or more PPCS that lasted for ≥1 month, but <9 months, after injury and to speak English. Adolescents with spinal cord or other severe injuries that had the potential to prevent participation were excluded as were those who had a diagnosis of schizophrenia or psychosis, or who presented with acute suicidal ideation. Adolescents with a history of previous concussion and/or other pre-existing psychological disorders were considered eligible. Parent-adolescent dyads were recruited between March 2017 and May 2019, and baseline data were collected during this period.

Of the 390 eligible adolescents screened, 80 actively declined to participate, and parents gave the following reasons: “too busy” (*n* = 33); “not interested” (*n* = 31); negative life event recently experienced (*n* = 1); and unknown (*n* = 15). An additional 109 parents did not respond to research coordinator contacts, thus passively declining, and one family was excluded from the study because we were unable to obtain their written consent. This resulted in a final sample size of 200 parent-adolescent dyads. Informed consent and assent were obtained before data collection, and study procedures were approved by the Seattle Children's Research Institute Institutional Review Board (protocol no.: STUDY00000437). The study was prospectively registered at ClinicalTrials.gov (NCT03034720). The findings presented herein are from adolescent- and parent-report surveys before randomization.

### Measures

At baseline, parents reported information on their child's age, race, ethnicity, insurance type (Medicaid or private), and socioeconomic status (parental education and household income). Adolescents reported on their gender identity. Parents reported on their adolescents' history of anxiety, depression, and any diagnosis of attention deficit hyperactivity disorder or attention deficit disorder. Youth trauma history was based on adolescent self-report (yes = 1, no = 0), on the 10-item Post-Traumatic Stress Disorder–Reaction Index, about specific lifetime traumatic events (possible total score of 10).^25–27^

Adolescents reported on their anxiety during the past 2 weeks using the GAD-7 (α = 0.87; 0 = not at all; 1 = several days; 2 = over half the days; 3 = nearly every day).^22^ Past research suggests that, for adolescents, a clinical cut point of 11 optimizes sensitivity and specificity for a diagnosis of “moderate anxiety” among youth with GAD.^18^ The 15-item anxiety subscale of the RCADS^28^ was used to assess adolescent report and parent report on current adolescent anxiety symptoms (α = 0.86 and 0.79, respectively). Participants rated the frequency of present anxious behaviors on a 4-point scale (0 = never; 1 = sometimes; 2 = often; and 3 = always). No clinical cutoff has been published for this measure. The anxiety subscale of the RCADS^28^ has been shown to be a reliable, valid measure of child and adolescent anxiety in general and clinical populations.^29^ Its inclusion in this study provides an opportunity to assess the convergent validity of the GAD-7.

Adolescent depressive symptoms were assessed using adolescent self-report with the 9-item Patient Health Questionnaire (PHQ-9; α = 0.84).^30,31^ Participants rated their symptoms over the past 2 weeks on a 4-point scale (0 = not at all; 1 = a little of the time; 2 = more than half the time; and 3 = nearly all the time). Reliability and validity of the PHQ-9 has been established in pediatric populations.^32^ Research indicates that, for adolescents, a clinical cut point of ≥11 optimizes sensitivity and specificity for detecting major depression.^32,33^ As a measure, the PHQ-9 can be used to assess the construct validity of the GAD-7.

### Procedure

Research assistants conducted participant outreach and enrollment and collected the baseline data. All data were collected using Research Electronic Data Capture—a secure, web-based application^34^—during the interview.

### Statistical analysis

With one exception, all statistical analyses were conducted using SAS software (version 9.4; SAS Institute Inc., Cary, NC).^35^ Three goodness-of-fit indices for the confirmatory factor analysis (CFA) models were estimated using the standard error of the mean command in STATA software (version 16; StataCorp LLC, College Station, TX),^36^ specifically, the 1) root mean square error of approximation (RMSEA) and its 90% confidence interval (CI), 2) comparative fit index (CFI), and 3) Tucker-Lewis index (TLI). For these analyses, we used the guidelines described herein: for the absolute fit index (RMSEA), excellent fit = 0.01–0.04, good fit = 0.05–0.07, mediocre fit = 0.08–0.10, and poor fit = >0.10; for the relative fit indices (CFI and TLI), excellent fit = 0.95–0.99, acceptable fit = 0.90–0.95, mediocre fit = 0.85–0.90, and poor fit = <0.85.^37-39^ Multiple indices were used because they provide different information for assessing model fit (i.e., fit adjusting for model parsimony, fit relative to a null model). When used together, these indices provide a more conservative, reliable evaluation of model fit.^38^ For tests of differences, *p* values were adjusted to account for multiple comparisons using the Hochberg and Hommel adjustments.^40^ Statistical significance was established at *p* < 0.05, and all tests were two-tailed.

## Results

Table 1 summarizes the characteristics of the study participants (adolescent *M*_age_ = 14.7 years, standard deviation [SD] = 1.7). Adolescent self-reported gender identity was 59% female, 37% male, and 1% transgender. The remaining youth (3.5%) did not respond to this question (*n* = 6) or selected, “I do not wish to answer” (*n* = 1). Parents reported that 82% of adolescents were white and 67% from households with annual incomes >$100,000. Parent respondents were mostly female (83%), mothers (81%), and fathers (17%), and 60% had bachelor's degrees or higher. In this sample, 50 adolescents (25%) had GAD-7 scores >10, which reflects moderate anxiety symptoms. Further, 79 adolescents (39.5%) had PHQ-9 scores >10, which is indicative of major depressive symptoms.

**Table 1.** Characteristics of the Adolescent and Parent Study Participants (*N* = 200)

	Adolescent	Parent
	No. (%*^a^*)	No. (%)
Gender		
Female	117 (58.5)	165^b^ (82.5)
Male	74 (37.0)	35 (17.5)
Transgender	2 (1.0)	
Unknown	7 (3.5)	
Age (years), mean (SD)	14.7 (1.7)	
Race		
American Indian or Alaska Native	1 (0.5)	
Asian	7 (3.5)	
Black	5 (2.5)	
More than one race	17 (8.5)	
Native Hawaiian or other Pacific Islander	1 (0.5)	
Other	3 (1.5)	
White	164 (82.0)	
Unknown	2 (1.0)	
Ethnicity		
Hispanic	17 (8.5)	
Non-Hispanic	177 (88.5)	
Unknown	6 (3.0)	
Parent respondent relation to adolescent		
Mother		162 (81.0)
Father		34 (17.0)
Grandmother		3 (1.5)
Guardian		1 (0.5)
Parent education		
High school graduate or less		13 (6.5)
Some college, no degree		35 (17.5)
Associate degree		32 (16.0)
Bachelor's degree		73 (36.5)
Graduate degree		46 (23.0)
Other		1 (0.5)
Other parent education		
High school graduate or less		30 (15.0)
Some college, no degree		30 (15.0)
Associate degree		27 (13.5)
Bachelor's degree		65 (32.5)
Graduate degree		43 (21.5)
Other		5 (2.5)
Annual household income		
< = $50K		17 (8.5)
$50–100K		38 (19.0)
>$100K		133 (66.5)
Unknown		12 (6.0)
Health insurance		
Medicaid		25 (12.5)
Private		168 (84.0)
Other		7 (3.5)
Adolescent anxiety symptoms (GAD-7), mean (SD)	7.24 (5.15)	
Adolescent anxiety symptoms (RCADS), mean (SD)		
Adolescent self-report	8.39 (6.75)	
Parent report on adolescent	5.22 (3.99)	
Adolescent depressive symptoms (PHQ-9), mean (SD)	9.68 (5.77)	

^a^ Percent unless otherwise noted.

^b^ Number of all female parents in the study.

GAD-7, Generalized Anxiety Disorder 7-item Scale; (GAD-7); RCADS, Revised Child Anxiety and Depression Scale-Short Version (anxiety subscale); PHQ-9, Patient Health Questionnaire-9 (PHQ-9); SD, standard deviation.

Characteristics of the GAD-7 items, including the corrected item-total correlations and factor loadings, are shown in Table 2. At the item level, the lowest mean was for item 7, “feeling afraid as if something awful might happen” (*M* = 0.60, SD = 0.89), and the second lowest mean was for item 5, “becoming so restless that it is hard to sit still” (*M* = 0.72, SD = 0.90). The highest mean was for item 6, “becoming easily annoyed or irritated” (*M* = 1.56, SD = 1.05). Items 5 and 6 had the lowest corrected item-total correlations (*r*_item 5_ = 0.54 and *r*_item 6_ = 0.51), which suggests that they were not as internally consistent with the other items. Aligned with these findings, the factor loadings from the CFA for items 5 and 6, at 0.56 and 0.53 respectively, were the lowest of the seven items. The loadings for the other five items were somewhat comparable and ranged from 0.66 to 0.83, indicating satisfactory-to-strong association with the factor.

**Table 2.** Characteristics of Items and Total Generalized Anxiety Disorder Scale (GAD-7)

No.	Item	Mean	SD	Corrected item-total correlation^a^	CFA factor loading
1	Feeling nervous, anxious or on edge	1.31	0.97	0.71	0.77
2	Not being able to stop or control worrying	0.92	1.01	0.75	0.83
3	Worrying too much about different things	1.13	1.04	0.76	0.83
4	Trouble relaxing	1.01	0.98	0.68	0.71
5	Being so restless that it is hard to sit still	0.72	0.90	0.54	0.56
6	Becoming easily annoyed or irritated	1.56	1.05	0.51	0.53
7	Feeling afraid as if something awful might happen	0.60	0.89	0.61	0.66
	GAD-7 total sum score	7.24	5.15	—	—

GAD-7 Cronbach's alpha = 0.87.

^a^ Correlation between the respective item and the total scale score (without the respective item).

GAD-7, General Anxiety Disorder 7-Item Scale; CFA, Confirmatory factor analysis; SD, standard deviation.

CFA fit indices for the one- and two-factor solutions are displayed in Table 3. For the one-factor solution, the RMSEA was 0.079 (90% CI, 0.041–0.116), which indicates mediocre fit. The CFI was 0.972 and the TLI was 0.958, which provides evidence of excellent fit. For the two-factor solution, the RMSEA was 0.121 (90% CI, 0.075–0.170), indicating a poor fit. The CFI was 0.966 and the TLI was 0.899, suggesting an excellent-to-acceptable fit. The one-factor model accounted for 50.75% of the variance whereas the two-factor model accounted for 58.44% of the variance, further supporting a unidimensional factor. Cronbach's alpha for scale was 0.87, which suggests high internal consistency (good reliability). When item 5 or item 6 was removed from the analysis, Cronbach's alpha remained unchanged at 0.87, indicating that high internal consistency is also achieved without these items.

**Table 3.** Confirmatory Factor Analysis Fit Indices for One- and Two-Factor Models of the Generalized Anxiety Disorder Scale (GAD-7)

	χ^2^	Df	RMSEA	RMSEA 90% CI	CFI		TLI
Model							
One-factor	613.10^***^	21	0.079	0.041–0.116	0.972		0.958
Two-factor	613.10^***^	21	0.121	0.075–0.170	0.966		0.899

For the absolute fit index (RMSEA), excellent fit = 0.01–0.04, good fit = 0.05–0.07, mediocre fit = 0.08–0.10, and poor fit = >0.10. For relative fit indices (CFI and TLI), excellent fit = 0.95–0.99, acceptable fit = 0.90–0.95, mediocre fit = 0.85–0.90, and poor fit = <0.85.

^***^ *p* < 0.001.

GAD-7, General Anxiety Disorder 7-Item Scale; CI, confidence interval; CFI, comparative fit index; *Df*, degrees of freedom; RMSEA, root mean square error of approximation; TLI, Tucker-Lewis index.

Correlation between adolescents' self-report of their anxious symptoms, as measured by the GAD-7 and the RCADS, was strong (*r* = 0.73, *p* < 0.001; Table 4). Magnitude of the correlation between adolescent self-report on the GAD-7 and parent-report of their child's anxiety symptoms on the RCADS was moderate (*r* = 0.29, *p* < 0.001) and consistent with literature on the correspondence between parent and adolescent ratings of adolescent internalizing symptoms.^41,42^ Correlation between adolescent self-report on the GAD-7 and the PHQ-9 was also robust (*r* = 0.77, *p* < 0.001).

**Table 4.** Means, Standard Deviations, Ranges, and Intercorrelations Between Anxiety and Depression Variables

	1	2	3	4
1. GAD-7				
2. RCADS (AR)	0.73^***^			
3. RCADS (PR)	0.29^***^	0.46^***^		
4. PHQ-9	0.77^***^	0.60^***^	0.18^*^	
Mean	7.24	8.39	5.22	9.68
SD	5.15	6.75	3.99	5.77
Range	0–21	0–34	0–23	0–26

^*^ *p* < 0.05.

^**^ *p* < 0.01.

^***^ *p* < 0.001.

AR, adolescent report; GAD-7, Generalized Anxiety Scale-7; PHQ-9, Patient Health Questionnaire-9; PR, parent report on adolescent; RCADS, Revised Child Anxiety and Depression Scale–Short Version (anxiety subscale); SD, standard deviation.

Finally, we examined mean differences for GAD-7 scores among key subpopulations (Table 5). We found statistically significant differences across all groups. Specifically, higher anxiety was reported by females and older adolescents. Youth with a history of anxiety, other mental health problems, trauma, and current depressive symptoms also experienced greater anxiety than their peers without these mental health characteristics. With one exception (for the gender unknown category), there was relatively little variation among Cronbach's alphas, which ranged from 0.76 to 0.89.

**Table 5.** Characteristics of the Generalized Anxiety Scale (GAD-7) Total Scores by Key Demographic, Psychological, and Stress Factors

	Mean	SD	Median	Alpha	Group difference,* p *value
Gender					**0.001** ^a^
Female (*n* = 117)	8.28	5.32	8.00	0.87	
Male (*n* = 74)	5.41	4.41	4.00	0.86	
Transgender (*n* = 2)	12.50	7.78	12.50	0.91	
Unknown (*n* = 7)	7.71	3.55	8.00	0.44	
Age, years					**0.034**
11–13 (*n* = 47)	6.34	5.39	5.00	0.89	
14–15 (*n* = 81)	6.65	4.74	6.00	0.86	
16–18 (*n* = 72)	8.49	5.25	8.00	0.86	
Past anxiety symptoms^b^					**0.004**
No (*n* = 129)	6.29	4.81	5.00	0.86	
Yes (*n* = 62)	8.81	5.13	8.00	0.87	
Past mental health problems^c^					**0.012**
0 (*n* = 152)	6.67	5.20	6.00	0.87	
≥1 (*n* = 48)	9.04	5.19	9.00	0.85	
Trauma event history^d^					**0.016**
0 or 1 (*n* = 135)	6.57	5.20	5.00	0.89	
≥2 (*n* = 65)	8.63	4.77	8.00	0.80	
Current depressive symptoms^e^					**<.0001**
No (*n* = 121)	4.65	3.35	4.00	0.76	
Yes (*n* = 79)	11.20	4.90	11.00	0.80	

*p* values that met statistical significance of *p* < 0.05 are in bold.

^a^ Because of the small sample size of participants who 1) identified as transgender (*n* = 2), 2) selected “I do not wish to answer” (*n* = 1), and 3) did not answer the gender identity question (*n* = 6), a group difference test was only conducted for the youth who identified as female or male.

^b^ Determined by parent responses about whether they had ever been told their child had anxiety problems.

^c^ Count variable determined by parent responses to individual items about whether they had ever been told their child had past health problems defined as 1) depression, 2) other mental health problems, or 3) whether their child had ever received a diagnosis of attention deficit hyperactivity disorder or attention deficit disorder before their injury.

^d^ Determined by the number of events endorsed on the trauma history portion of the Post-Traumatic Stress Disorder–Reaction Index (PTSD-RI), UCLA Trauma History Profile.

^e^ Determined by adolescent responses to the Patient Health Questionnaire-9.

GAD-7, General Anxiety Disorder 7-Item Scale; SD, standard deviation.

## Discussion

The evidence base for GAD-7 as a measure with clinical utility and strong psychometric properties in youth is emerging, and this article demonstrates similar properties among adolescents with PPCS. Overall, our results indicate that a one-factor structure of the GAD-7 was supported by CFA, aligning with Tiirikainen and colleagues' results for adolescents, which was also in support of unidimensionality. Whereas the RMSEA goodness-of-fit indices from the factor analyses were in the “mediocre” range, both the CFI and TLI indicators, which are less sensitive to sample size, suggested excellent fit. Convergent validity was supported by the findings, which suggest that higher GAD-7 scores were associated with higher levels of self-reported depressive symptoms and self- and parent report of anxiety symptoms using the RCADS. These findings are consistent with results from a large, representative study of Finnish adolescents that revealed good psychometric properties for the GAD-7, with nearly identical Cronbach's alphas at 0.9^19^ and a separate validation study based on 40 adolescents with GAD.^18^

At the same time, items 5 (restlessness) and 6 (irritability) had the lowest corrected item-total correlations and factor loadings. These findings are also aligned with Tiirikainen and colleagues' study, in which these items had the lowest corrected item total correlation and factor loadings.^19^ In terms of face validity, these items tap broader psychopathology symptoms that are not specific to anxiety, but also common to depression and attention disorders. Further, Cronbach's alpha remained unchanged in our study when these two items were removed, indicating that they do not contribute to the internal validity of the measure. A study by Moreno and colleagues with adult primary care patients in Spain who presented with internalizing disorders (*N* = 1255) found that a two-factor model (representing cognitive-emotional and somatic components of anxiety) provided a superior fit compared to the one-factor model. Specifically, items 5 and 6 in tandem with item 4 (trouble relaxing) comprised the somatic dimension, and items 1, 2, 3, and 7 (i.e., “1. feeling nervous, anxious or on edge;” “2. not being able to stop or control worrying;” “3. worrying too much about different things;” and “7. feeling afraid as if something awful might happen”) comprised the cognitive-affective dimension.^43^ This same pattern of results was obtained in two studies with adult psychiatric samples.^44,45^ Notably, in the current study, three of the items that comprised this cognitive affective dimension (1, 2, and 3) also had the highest factor loadings. A study by Jordan and colleagues with adult German primary care patients suggested that the first four GAD-7 items discriminated anxiety severity better than the last three items.^21^ These findings, considered together with ours, may indicate that the GAD-7 items that tap more physically focused complaints (items 4, 5, and 6), such as restlessness, may be secondary to emotion-focused items (items 1, 2, 3, and 7) among adolescents with PPCS.

With respect to our aim to understand whether differences exist for GAD-7 scores among key subpopulations, differences were detected for each factor examined and consistent with other studies, demonstrating higher anxiety symptoms among adolescent females, older youth, and adolescents with current depressive symptoms.^19^ Not surprisingly, adolescents with a history of anxiety, other mental health problems, and trauma also reported greater anxiety than their counterparts without these mental health characteristics. We believe that these findings strengthen the evidence for the measure's construct validity considering that adolescents with higher anxiety levels, mental health challenges, and trauma histories scored higher on the GAD-7. For nearly all subgroups, Cronbach's alphas indicated acceptable-to-excellent internal consistency. The one exception to this was for the unknown gender category, which had a small sample size (*n* = 7).

### Limitations

There are several study limitations. Our sample was primarily white (82%) and non-Hispanic (89%), limiting generalizability to other racial and ethnic groups. These findings are further constrained by the higher proportion of females relative to males and the lack of socioeconomic diversity (adolescents were predominantly from households with high incomes and parent education). Recent research documents disparities among adolescents who present for concussion care, with non-Hispanics and those with private insurance more likely to have healthcare visits.^46^ Thus, our sample may be representative of adolescents who present for concussion care, but it also may be limited in generalizability for all who experience PPCS. Finally, the small number of youth who identified as transgender or who opted out of providing this information further curtails the utility of these findings for adolescents who may identify as gender non-binary and are likely at higher risk for anxiety and depression.^47^

## Conclusion

Our results suggest that the GAD-7 is a valid screening tool among youth experiencing PPCS and that it is a unidimensional factor. Scores are strongly correlated with youth self-report of depressive and anxious symptoms on other scales and moderately correlated with parental report, indicating the importance of obtaining youth-reported symptoms, which may not always be observed by parents. Adolescents with previous anxiety, trauma, and mental health symptoms scored higher on the GAD-7 compared to those without, as did those with current depression. Further research should explore whether the inclusion of items assessing restlessness and irritability adds to the validity of the GAD-7, or whether these non-specific items could be removed. Nevertheless, it offers a means for primary care providers and other clinicians caring for adolescents with concussion to more fully ascertain the full spectrum of problems encountered after concussion.

## Abbreviations Used

CFA: confirmatory factor analysis
CFI: comparative fit index
CI: confidence interval
GAD: generalized anxiety disorder
GAD-7: General Anxiety Disorder 7-Item
PHQ-9: Patient Health Questionnaire-9
PPCS: persistent post-concussive symptoms
RCADS: Revised Child Anxiety and Depression Scale–Short Version
RMSEA: root mean square error of approximation
SD: standard deviation
TLI: Tucker-Lewis index
